# Association between Physical Function at Discharge and Fall Frequency One Month after Discharge in Patients with Progressive Supranuclear Palsy

**DOI:** 10.1298/ptr.25-E10361

**Published:** 2025-11-22

**Authors:** Yutaro SATO, Hideki YOSHIDA, Keisuke OTA, Kyoko HAMADA, Naoto ISHIKAWA, Ryo MATSUDA

**Affiliations:** 1 Department of Rehabilitation, Neuroscience Research Center Shin Sapporo Neurosurgical Hospital, Japan; 2 Graduate School of Health Sciences, Hirosaki University, Japan; 3 Department of Rehabilitation, Hokuyu-kai Neurological Hospital, Japan; 4 Department of Neurology, Neuroscience Research Center Shin Sapporo Neurosurgical Hospital, Japan; 5 Department of Physical Therapy, Faculty of Health Sciences, Hokkaido University of Science, Japan

**Keywords:** Progressive Supranuclear Palsy, Fall frequency, Balance function, Anticipatory postural adjustments

## Abstract

**Objectives:**

The aim of this study was to investigate the relationship between fall frequency and progressive supranuclear palsy (PSP) severity, walking ability, balance function, cognitive function, and frontal lobe function in patients with PSP.

**Methods:**

The study included 54 patients with PSP. Multiple logistic regression analysis was conducted to examine whether PSP severity, walking ability, balance function, cognitive function, and frontal lobe function after discharge were associated with fall frequency (falling two or more times per month). Balance function was assessed using the Berg Balance Scale and Mini-Balance Evaluation Systems Test (Mini-BESTest), with each used as an independent variable in separate models for association analysis. The Mini-BESTest showed a significant association, and its subitems were further analyzed using multiple logistic regression analysis.

**Results:**

A significant association was observed between the Mini-BESTest and fall frequency, and among its subitems, anticipatory postural adjustments were significantly associated with fall frequency.

**Conclusions:**

In patients with PSP characterized by impaired postural reflexes, the anticipatory postural adjustment ability may be associated with fall frequency.

## Introduction

Progressive supranuclear palsy (PSP) is a neurodegenerative disorder related to Parkinson’s disease (PD). It is characterized by supranuclear gaze palsy, postural instability, gait disturbance, pseudobulbar palsy, axial rigidity, and dementia^1,2)^. The diagnostic criteria for PSP include falling within the first year^3)^. Compared with other neurodegenerative diseases, PSP is characterized by a higher frequency of falls and an increased incidence of fractures due to falls^4,5)^. Therefore, fall propensity can be considered as one of the most characteristic symptoms of PSP that contributes to the decline in physical function.

Furthermore, since PSP progresses more rapidly and manifests more diverse symptoms compared with PD, interventions focusing on fall prevention strategies and treatment programs are essential from the early stages of the disease, both during hospitalization and after discharge. Additionally, studies have reported that patients with PSP who have high fall frequencies exhibit greater disease severity, decreased walking ability, balance function, cognitive function, and frontal lobe function^5–7)^.

Other studies indicated that compared with patients with PD and older individuals, patients with PSP demonstrate decreased walking ability, balance function, cognitive function, and frontal lobe function^8–17)^.

Consequently, various functional factors may contribute to fall risk in patients with PSP compared with those with other neurodegenerative diseases. Although increased fall frequency is often observed in patients with PSP soon after discharge due to environmental changes in clinical settings, no studies have evaluated the relationship between fall frequency immediately after discharge and multiple physical function factors at discharge.

Therefore, the purpose of this study was to classify fall frequency within 1 month after discharge and determine which factors among PSP disease severity, gait function, balance function, cognitive function, and frontal lobe function at discharge could be associated with fall frequency.

Previous studies have reported that the Mini-Balance Evaluation Systems Test (Mini-BESTest) shows higher predictive accuracy for falls compared with the Berg Balance Scale (BBS) in patients with neurodegenerative diseases^18)^.

Falls in patients with PSP often occur suddenly in unpredictable situations^4)^ and may be associated with balance impairments during tasks that are not purely dynamic but closely related to daily life, such as maintaining a static posture, rising from a chair, or turning while standing. The BBS evaluates static and quasi-dynamic balance functions, including sitting balance, sit-to-stand movements, and turning while standing, which are difficult to assess by the Mini-BESTest, and thereby remains clinically useful for predicting falls and guiding interventions. In contrast, characteristic postural reflex impairments in PSP cases are directly linked to multifaceted and unpredictable falls, such as impaired backward stepping responses^5,19)^ and increased fall risk under dual-task conditions^7)^. The Mini-BESTest includes items that specifically assess these functions, such as postural responses and dual-task walking, and may consequently be more sensitive to detecting fall risk in patients with PSP. Therefore, although both the BBS and the Mini-BESTest are useful from different perspectives, we hypothesized that the Mini-BESTest score would be particularly associated with falls in patients with PSP.

## Methods

### Study design

This study employed a prospective cohort design. It was conducted in accordance with the Declaration of Helsinki and approved by the Ethics Committee of New Sapporo Neurosurgical Hospital, Medical Corporation Brain Research Center (approval date: February 16, 2021) and the Ethics Committee of Hirosaki University Graduate School of Health Sciences (approval number: 2023-007). The purpose and measurement details of this study were explained to all participants, and written informed consent was obtained from each participant.

### Participants and methods

**Fig. 1.**
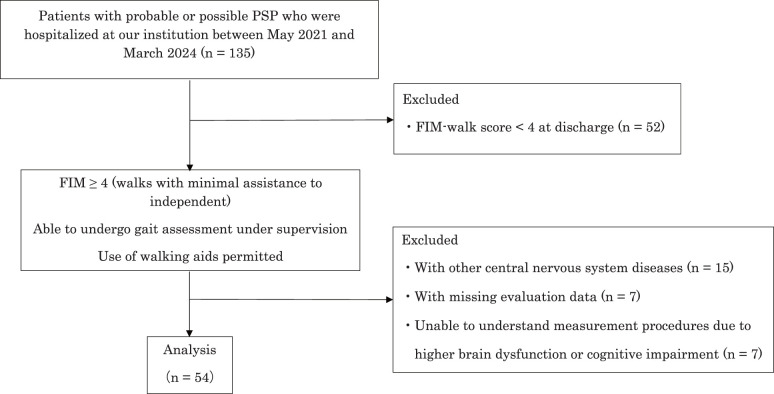
Flow diagram of patient enrollment and exclusion criteria PSP, progressive supranuclear palsy; FIM, functional independence measure

We identified 135 patients diagnosed with probable PSP or possible PSP according to the Movement Disorder Society Clinical Diagnostic Criteria for PSP (MDS PSP criteria)^20)^ who were hospitalized at our institution between May 2021 and March 2024^19,21)^. Of these, we excluded: (1) 52 patients with Functional Independence Measure walking item scores less than 4, (2) 15 patients with other central nervous system diseases, (3) 7 patients with missing evaluation data, and (4) 7 patients who had difficulty understanding the measurement procedures due to higher brain dysfunction or cognitive impairment. This resulted in a final sample of 54 participants (Fig. 1).

### Assessment items

The assessment items included age, sex, body mass index (BMI), disease duration, PSP subtype^20)^, Hoehn and Yahr scale (H&Y scale)^22)^, lower limb muscle strength^23,24)^, and Push and Release Test (P&R test)^25)^. They also comprised the Pull test^26)^, Progressive Supranuclear Palsy Rating Scale total (PSPRS total)^27)^, PSPRS subscales (history, mentation, bulbar, ocular motor, limb motor, gait, and midline), comfortable walking speed (CWS)^28)^, BBS^29)^, Mini-BESTest total^30)^, and Mini-BESTest subscales (anticipatory postural adjustments, postural responds, sensory orientation, and balance during gait). The Mini-Mental State Examination (MMSE)^31)^ and Frontal Assessment Battery (FAB) were also assessed^32)^.

PSP subtypes were classified by the neurologists at the hospital according to the MDS PSP criteria^20)^ into PSP-Richardson’s syndrome (PSP-RS), PSP-progressive gait freezing (PSP-PGF), PSP-predominant parkinsonism (PSP-P), and PSP-predominant speech/language disorder.

The PSPRS^27)^, which evaluates PSP severity, consists of 6 sessions with 28 items across history, mental symptoms, bulbar symptoms, ocular symptoms, limb symptoms, and gait/trunk symptoms, with a total score of 100 points. Higher scores indicate greater severity. PSPRS evaluations were performed according to the Japanese translation definitions^33)^.

The assessment of lower limb muscle strength was performed by measuring knee extensor strength^23)^. Knee extensor strength was measured using a hand-held dynamometer (μ-Tas F-1; ANIMA, Tokyo, Japan) with the H-stabilization method^23)^, following previously reported procedures. Measurements were performed twice on each side, and the maximum value was used for analysis^24)^. The measured values were converted into knee extensor strength relative to body weight (kgf/kg). This variable was included as a covariate in the logistic regression analysis.

CWS^28)^ was measured on a 16-m straight path with 3-m acceleration and deceleration zones at both ends of a 10-m measurement zone. The participants were instructed to “walk as usual” and use their daily assistive devices. After sufficient practice, CWS was measured twice using a stopwatch, and the average of the two measurements was used for analysis^28)^.

The BBS^29)^ evaluates balance function through 14 tasks scored: 0–4 points, with a total maximum score of 56 points. Higher scores indicate better balance ability.

The Mini-BESTest^29)^ consists of 4 sessions (anticipatory postural adjustments, postural responses, sensory orientation, and balance during gait) with 14 tasks scored from 0 to 2 points, with a total maximum score of 28 points. Higher scores indicate better balance function.

The P&R test and Pull test are balance function evaluations scored from 0 to 4 points, with lower scores indicating better balance function. The P&R test and Pull test were conducted according to methods described in previous studies^25,26)^. All outcome measures were assessed at the time of discharge.

### Assessment of fall incidence

Fall frequency was evaluated 1 month after discharge. A fall was defined as “unintentionally stumbling or slipping during walking or activity, resulting in contact of a body part such as the hand or buttocks with the floor, ground, or a lower position”^34)^. Fall frequency was assessed by interviewing cohabitating family members or the patients themselves at outpatient visits or by telephone survey 1 month after discharge to investigate the frequency of falls during that month.

To reduce recall bias, verbal instructions were given to cohabitating family members or patients to record falls daily. Based on previous research, participants who experienced multiple falls (two or more falls) within 1 month were classified as the high fall risk group, while those who experienced one fall or no falls were classified as the low fall risk group^7)^.

### Statistical analysis

Regarding patient characteristics between the high fall risk and low fall risk groups, categorical variables were analyzed using the chi-squared test, while other variables were analyzed using the independent t-test or the Mann–Whitney U test.

To examine which variables affected fall frequency, multiple logistic regression analysis was performed with the high fall risk and low fall risk groups as dependent variables (forced entry method), with the high-risk group coded as 1 and the low-risk group coded as 0. The independent variables included PSP severity assessment (PSPRS total), gait function (CWS), balance function (BBS, Mini-BESTest), cognitive function (MMSE), and frontal lobe function (FAB), which were considered clinically possible to be associated with fall risk. For the balance function, the BBS and Mini-BESTest were included as independent variables in separate models. After analyzing the relationship with the PSPRS total, CWS, BBS, MMSE, and FAB (BBS Model 1), confounding factors (age, sex, BMI, disease duration, H&Y scale, and knee extensor strength relative to body weight) were progressively added (BBS Model 2). Similarly, after evaluating the relationship with the PSPRS total, CWS, Mini-BESTest, MMSE, and FAB (Mini-BESTest Model 1), confounding factors were progressively added (Mini-BESTest Model 2) to assess the relationship between fall frequency 1 month after discharge and physical function.

Regarding the multiple logistic regression analysis using the Mini-BESTest subscales (anticipatory postural adjustments, postural responses, sensory orientation, and balance during gait), two models were constructed. In Model 1, only the Mini-BESTest subscales were included. In Model 2, confounding factors (age, sex, BMI, disease duration, H&Y scale, and knee extensor strength relative to body weight) were progressively added as covariates.

Additionally, variance inflation factor values were calculated, and variables with values of 10 or higher were excluded from the independent variables^35)^. All statistical analyses were performed using R statistical software (version 4.2.1; R Foundation for Statistical Computing, Vienna, Austria), with a significance level of 5%.

## Results

### Characteristics of high fall risk group and low fall risk group

**Fig. 2.**
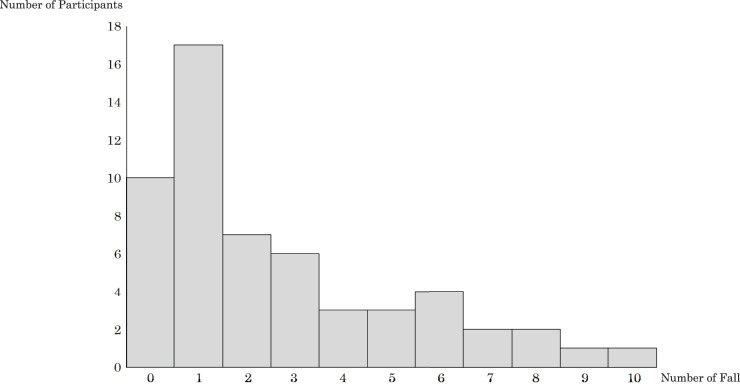
Distribution of fall frequency

The distribution of fall frequencies is shown in Figure 2. The mean ± standard deviation of falls in the entire group was 2.6 ± 2.5 falls, with a median (interquartile range) of 2.0 (1–10) falls. Table 1 presents the basic characteristics and assessment items for each group. Regarding categorical variables, significant differences between the high fall risk and low fall risk groups were found in the H&Y scale (p <0.001), P&R test (p <0.001), and Pull test (p = 0.001). For other variables, compared with the low fall risk group, the high fall risk group showed significantly longer disease duration (p = 0.001), higher PSPRS total (p = 0.001), and higher PSPRS subscales including ocular motor (p = 0.010), and gait and midline (p <0.001). In contrast, the high fall risk group showed significantly lower values for CWS (p <0.001), BBS (p <0.001), Mini-BESTest total (p <0.001), and Mini-BESTest subscales including anticipatory postural adjustments (p <0.001), postural responses (p <0.001), sensory orientation (p <0.001), and balance during gait (p <0.001).

**Table 1.** Patient characteristics by fall frequency group

	All participants (n = 54)	Fall frequency 1 month after discharge		p-Value
		Low fall risk group (n = 26)	High fall risk group (n = 28)	
Age (years)	78.8 ± 7.6	77.0 ± 7.0	80.4 ± 7.9	0.100
Sex (%)				0.586
Female	27 (50.0)	14 (51.2)	13 (48.8)	
Male	27 (50.0)	12(44.4)	15 (53.6)	
BMI (kg/m^2^)	20.7 ± 3.0	20.8 ± 2.9	20.5 ± 3.1	0.588
Disease duration (months)	36.9 ± 28.6	27.2 ± 19.1	47.5 ± 33.1	0.001
PSP subtype (%)				
PSP-RS	26 (48.1)	8 (30.7)	19 (63.3)	
PSP-PGF	23 (51.9)	15 (57.7)	9 (30.1)	
PSP-P	3 (5.4)	2 (7.7)	1 (3.3)	
PSP-SL	2 (3.6)	1 (3.9)	1 (3.3)	
Hoehn and Yahr scale (%)				<0.001
Stage III	34 (62.5)	25 (73.5)	9 (22.5)	
Stage IV	20 (37.5)	1 (5.0)	19 (95.0)	
Knee extension strength-to-body weight ratio (kgf/kg)	0.3 ± 0.1	0.4 ± 0.1	0.3 ± 0.1	
PSPRS total	17.6 ± 11.3	12.6 ± 7.3	22.2 ± 12.4	0.001
PSPRS history	4.4 ± 3.0	3.8 ± 2.2	5.0 ± 3.5	0.126
PSPRS mentation	1.7 ± 2.2	1.2 ± 1.6	2.3 ± 2.6	0.124
PSPRS bulbar	0.7 ± 1.3	0.3 ± 0.5	1.0 ± 1.6	0.169
PSPRS ocular motor	2.5 ± 2.9	1.5 ± 2.4	3.4 ± 3.2	0.010
PSPRS limb motor	2.8 ± 1.5	2.7 ± 1.6	3.0 ± 1.3	0.398
PSPRS gait and midline	5.4 ± 3.9	3.2 ± 1.4	7.5 ± 4.3	<0.001
Pull test	2.4 ± 0.7	2.1 ± 0.7	2.8 ± 0.5	0.001
P&R test	2.2 ± 1.3	1.4 ± 1.2	2.9 ± 1.0	<0.001
CWS (m/sec)	0.7 ± 0.2	0.8 ± 0.2	0.6 ± 0.2	<0.001
Mini-BESTest total	14.3 ± 7.0	19.3 ± 3.9	9.6 ± 5.8	<0.001
Mini-BESTest anticipatory postural adjustments	3.1 ± 1.8	4.4 ± 1.1	1.9 ± 1.5	<0.001
Mini-BESTest postural responses	2.2 ± 1.9	3.5 ± 1.5	1.0 ±1.4	<0.001
Mini-BESTest sensory orientation	3.8 ± 1.8	4.8 ± 1.2	2.9 ± 1.7	<0.001
Mini-BESTest balance during gait	5.2 ± 2.4	6.7 ± 1.6	3.8 ± 2.2	<0.001
BBS	44.1 ± 10.3	50.0 ± 4.5	38.5 ± 11.1	<0.001
MMSE	24.8 ± 4.4	24.3 ± 4.2	23.9 ± 5.1	0.240
FAB	13.0 ± 3.6	14.0 ± 3.2	12.1 ± 3.8	0.059

BMI, body mass index; PSP, Progressive Supranuclear Palsy; PSP-RS, PSP Richardson’s syndrome; PSP-PGF, PSP progressive gait freezing; PSP-P, PSP predominant parkinsonism; PSP-SL, PSP predominant speech language disorder; PSPRS, Progressive Supranuclear Palsy Rating Scale; P&R test, Push and Release Test; CWS, comfortable walking speed; Mini-BESTest, Mini-Balance Evaluation Systems Test; BBS, Berg Balance Scale; MMSE, Mini Mental State Examination; FAB, Frontal Assessment Battery

### Factors associated with fall frequency

In the BBS model, the BBS total was significantly associated with fall frequency in Model 1 (odds ratio: 0.85, 95% confidence interval: 0.70–1.00, p = 0.04). However, in Model 2 with confounding factors, no variable was found as a significant factor (odds ratio: 0.87, 95% confidence interval: 0.69–1.05, p = 0.18), indicating that higher BBS scores were related to a lower frequency of falls only in Model 1 (Table 2). In the Mini-BESTest model, significant associations were observed for the Mini-BESTest total in both Model 1 (odds ratio: 0.64, 95% confidence interval: 0.44–0.82, p = 0.01) and Model 2 with confounding factors (odds ratio: 0.68, 95% confidence interval: 0.43–0.91, p = 0.042) (Table 3). Among the Mini-BESTest subscale models, anticipatory postural adjustments were significantly associated with fall frequency in Model 1 (odds ratio: 0.33, 95% confidence interval: 0.11–0.75, p = 0.02). However, in Model 2 with confounding factors, no significant factors were found (odds ratio: 0.34, 95% confidence interval: 0.09–0.89, p = 0.06), indicating that anticipatory postural adjustments were related to a lower frequency of falls only in Model 1 (Table 4).

**Table 2.** Multiple logistic regression analysis using the BBS model with fall frequency as the dependent variable

	Model 1					Model 2				
	Regression coefficient	p-Value	OR	95% CI	VIF	Regression coefficient	p-Value	OR	95% CI	VIF
BBS	−0.17	0.04	0.85	0.70–1.00	1.52	−0.14	0.18	0.87	0.69–1.05	1.98
PSPRS total	−0.01	0.97	1.00	0.91–1.10	1.39	−0.09	0.27	0.91	0.75–1.05	3.47
CWS	−3.31	0.18	0.04	0–3.32	1.20	−3.68	0.25	0.03	0–8.27	1.39
MMSE	−0.07	0.60	0.93	0.71–1.21	1.73	−0.10	0.52	0.91	0.65–1.22	2.15
FAB	−0.07	0.61	0.93	0.69–1.24	1.64	−0.11	0.56	0.90	0.6–1.3	2.20
Age						0.05	0.44	1.06	0.92–1.23	1.60
Sex						0.49	0.63	1.64	0.22–13.17	1.66
BMI						−0.16	0.36	0.85	0.58–1.18	1.57
Disease duration						0.03	0.26	1.03	0.98–1.07	1.40
Hoehn and Yahr scale						2.64	0.06	14.01	1.19–373.92	2.17
Knee extension strength-to-body weight ratio						0.38	0.52	0.91	0.65–1.22	1.40

BBS, Berg Balance Scale; OR, odds ratio; 95% CI, 95% confidence intervall VIF, variance inflation factor; PSPRS; Progressive Supranuclear Palsy Rating Scale; CWS, comfortable walking speed; MMSE, Mini Mental State Examination; FAB, Frontal Assessment Battery; BMI, body mass index

**Table 3.** Multiple logistic regression analysis using the Mini-BESTest model with fall frequency as the dependent variable

	Model 1					Model 2				
	Regression coefficient	p-Value	OR	95% CI	VIF	Regression coefficient	p-Value	OR	95% CI	VIF
Mini-BESTest total	−0.45	0.01	0.64	0.44–0.82	2.05	−0.39	0.04	0.68	0.43–0.91	2.44
PSPRS	−0.09	0.21	0.92	0.79–1.04	2.03	−0.13	0.16	0.88	0.70–1.03	2.96
CWS	−3.56	0.24	0.03	0–7.54	1.10	−4.67	0.22	0.01	0–6.73	1.55
MMSE	−0.12	0.30	0.84	0–1.17	2.04	−0.16	0.38	0.86	0.58–1.19	2.25
FAB	0.01	0.95	1.01	0.72–1.42	1.79	−0.01	0.99	0.99	0.67–1.48	2.41
Age						0.03	0.75	1.03	0.87–1.21	1.66
Sex						−0.06	0.96	0.95	0.07–10.68	1.92
BMI						−0.22	0.38	0.81	0.46–1.25	1.79
Disease duration						0.02	0.39	1.02	0.97–1.08	1.43
Hoehn and Yahr scale						1.40	0.37	0.99	0.67–1.48	2.05
Knee extension strength-to-body weight ratio						0.83	0.85	2.28	0–25505.60	1.17

Mini-BESTest, Mini-Balance Evaluation Systems Test; OR, odds ratio; 95% CI, 95% confidence interval; VIF, variance inflation factor; PSPRS, Progressive Supranuclear Palsy Rating Scale; CWS, comfortable walking speed; MMSE, Mini Mental State Examination; FAB, Frontal Assessment Battery; BMI, body mass index

**Table 4.** Multiple logistic regression analysis of Mini-BESTest subscales with fall frequency as the dependent variable

	Model 1					Model 2				
	Regression coefficient	p-Value	OR	95% CI	VIF	Regression coefficient	p-Value	OR	95% CI	VIF
Anticipatory postural adjustments	−1.10	0.02	0.33	0.11–0.75	1.25	−1.09	0.06	0.34	0.09–0.89	1.44
Postural responses	−0.78	0.06	0.46	0.18–0.93	1.36	−0.75	0.10	0.47	0.16–1.07	1.62
Sensory orientation	−0.01	0.97	0.99	0.47–2.01	1.51	0.10	0.84	1.10	0.44–2.88	2.36
Balance during gait	0.06	0.87	1.06	0.51–2.17	1.74	0.16	0.70	1.17	0.52–2.70	2.22
Age						0.08	0.29	1.09	0.94–1.29	1.44
Sex						0.07	0.95	1.08	0.11–11.05	1.64
BMI						−0.08	0.70	0.94	0.61–1.41	1.38
Disease duration						0.01	0.78	1.01	0.96–1.06	1.35
Hoehn and Yahr scale						1.11	0.39	3.05	0.24–48.59	1.60
Knee extension strength-to-body weight ratio						−1.25	0.79	0.29	0–4319.68	1.28

Mini-BESTest, Mini-Balance Evaluation Systems Test; OR, odds ratio; 95% CI, 95% confidence interval; VIF, variance inflation factor; BMI, body mass index

## Discussion

This study longitudinally investigated the relationship between fall frequency 1 month after discharge and PSP disease severity, gait/balance function, and cognitive/frontal lobe function at discharge in patients with PSP.

The results showed significantly higher values in the high fall risk group compared with the low fall risk group for disease duration, H&Y scale, P&R test, Pull test, PSPRS total, and PSPRS subscales, including mentation, ocular symptoms, and gait/trunk symptoms. Significantly lower values were found for CWS, BBS, Mini-BESTest total, and Mini-BESTest subscales, including anticipatory postural adjustments, postural responses, sensory orientation, and balance during gait.

Multiple logistic regression analysis revealed that both the BBS total score and the anticipatory postural adjustments subscale of the Mini-BESTest were significantly associated with fall frequency in Model 1. However, they were not found to be significant factors in Model 2 after adjusting for confounding variables. In contrast, the Mini-BESTest total score was significantly associated with fall frequency in both Model 1 and Model 2. The following sections discuss the factors related to fall frequency 1 month after discharge and the relationship between the Mini-BESTest subscales and fall frequency.

### Factors related to fall frequency 1 month after discharge

In this study, the BBS and Mini-BESTest were included as independent variables in separate models for multiple logistic regression analysis, which revealed that the Mini-BESTest was significantly associated with fall frequency.

According to Sibley et al.^36)^, the BBS includes six balance components (stability limits, motor system, static postural stability, anticipatory postural adjustments, dynamic stability, and sensory orientation), while the Mini-BESTest includes eight balance components (motor system, static postural stability, anticipatory postural adjustments, dynamic stability, sensory orientation, verticality, postural responses, and cognitive influence). Thus, the Mini-BESTest encompasses more diverse balance components compared to BBS, allowing for a more multifaceted balance function assessment, which suggests its association with fall frequency.

However, the BBS includes tasks that are not covered by the Mini-BESTest, such as sitting balance, sit-down movements, transfers, picking up an object from the floor, and forward reaching, which are directly related to activities of daily living. Therefore, although the BBS did not emerge as an independent factor compared with the Mini-BESTest in predicting fall frequency, it appears to play a complementary role in clinical practice. This suggests that in patients with PSP who have a high frequency of falls, a combined use of both assessments may be necessary for comprehensively evaluating balance function.

In patients with PSP and other degenerative diseases such as PD and spinocerebellar degeneration, studies have reported that stepping ability assessments for external perturbation^19,37,38)^ and the Mini-BESTest^18,39)^ show higher predictive accuracy for walking independence and fall occurrence compared with BBS. This suggests that the Mini-BESTest, which consists of compensatory corrective steps in all directions and walking under dual-task conditions, may be more sensitive in detecting balance function decline compared with the BBS, which includes relatively low-difficulty tasks such as sitting, standing, and 360-degree rotation. On the other hand, general cognitive function and frontal lobe function assessments revealed no significant variables.

Longitudinal studies tracking PSPRS total scores^18)^ and general cognitive and frontal lobe function^40,41)^ in patients with PSP have reported that cognitive and frontal lobe functions decline more slowly compared with PSPRS total scores, which include limb/trunk function, gait function, and balance function assessments. Additionally, even when excluding patients with PSP with cognitive function decline, studies have found no significant relationship between fall frequency and frontal lobe function but a significant relationship between fall frequency and ocular motor symptoms, limb symptoms, and gait/trunk symptoms^6)^. These findings suggest that physical function aspects such as balance function may be more important than cognitive/frontal lobe function in relation to fall frequency in patients with PSP.

Furthermore, even when patients with cognitive decline were excluded, fall frequency was not associated with frontal lobe function but was significantly related to oculomotor, limb, and gait/trunk symptoms^6)^. This finding supports the notion that, regardless of cognitive status, body functions such as balance and gait play a greater role in falls in patients with PSP.

### Relationship between Mini-BESTest subscales and fall frequency 1 month after discharge

Previous studies have shown that anticipatory postural adjustments are modulated according to task and environmental conditions. Aruin et al.^42)^ reported that during upper limb movements while standing, anticipatory postural adjustments were observed in the trunk and lower limb muscles under stable conditions, whereas these activities were significantly attenuated under unstable conditions such as a feet-together stance. This suggests that the central nervous system suppresses anticipatory postural adjustments to prevent excessive postural instability.

In contrast, Weaver et al.^43)^ demonstrated that during tasks with a severely restricted base of support, such as unilateral stance, trunk muscle activity, including the rectus abdominis and external oblique, was facilitated. These findings indicate that both insufficient and excessive anticipatory postural adjustments may compromise postural stability and contribute to fall risk, emphasizing the importance of their appropriate modulation. The anticipatory postural adjustment items in the Mini-BESTest, such as rising on tiptoes and standing on one leg, require initiating movement under a narrow base of support. By reducing body sway and decreasing the need for compensatory responses, anticipatory postural adjustments may play a particularly crucial role in patients with PSP, who are characterized by impaired postural reflexes.

With regard to fall prevention, reducing body sway through anticipatory postural adjustment decreases the need for postural responses^44)^, suggesting that anticipatory postural adjustment ability is important for managing patients with PSP who have postural reflex disorders. Welter et al.^8)^ reported that in older individuals, postural control is maintained during both optimal and maximum walking to prevent excessive lowering of the center of gravity at heel contact, whereas patients with PSP show markedly decreased ability to control center of gravity lowering during maximum walking, increasing gait instability. Amano et al.^9)^ reported that in older individuals and patients with PD, the center of foot pressure during gait initiation moves toward the swing leg similar to normal gait, contributing to improved forward propulsion for gait initiation. However, in patients with PSP, the center of foot pressure moves toward the support side. These findings further suggest that patients with PSP have decreased anticipatory postural adjustment ability during walking compared with healthy older individuals and patients with PD, which may be associated with fall frequency.

However, postural response was not found to be a significant variable, differing from previous research^21)^. In this study, postural response ability in patients with PSP showed significant differences between the high and low fall risk groups; however, both groups demonstrated decline. Additionally, compared with other subscales (anticipatory postural adjustments, sensory orientation, and balance during gait), postural responses tended to show greater decline. Therefore, it appears that patients with PSP, who primarily exhibit postural reflex disorders, have insufficient postural control ability in response to external perturbation regardless of fall frequency.

Based on the results of this study, rehabilitation programs aimed at reducing fall frequency in patients with PSP may need to focus on balance interventions that include initial movement actions such as postural maintenance, sit-to-stand practice, and turning exercises from standing positions. Among these initial movements, individually evaluating those associated with particularly high fall risk in clinical settings and prioritizing training according to the evaluation results are important. Previous studies have shown that repetitive task practice is effective in anticipatory postural adjustments^45)^, and further investigation is needed to determine whether this is effective in reducing fall frequency in patients with PSP.

However, in the subscale analysis, anticipatory postural adjustments did not remain a significant independent factor when fitted as covariates. Therefore, implementing comprehensive balance interventions that include stepping responses to external perturbations and dual-task activities is necessary, and further studies are required to verify their effectiveness. Furthermore, since patients with PSP often experience sudden and unpredictable backward falls, close supervision, use of safety harnesses or parallel bars, and environmental adjustments are essential to minimize fall risk during interventions.

### Limitations and future directions

This study has several limitations. First, there is the issue of sample size. With 56 participants and multiple explanatory variables, further investigation with a larger sample is needed. However, this study provides valuable findings given that PSP is a rare disease.

Second, this study focused on fall frequency over a short period of 1 month after discharge, and the relationship between falls and physical function over a longer period remains unclear. Future studies should examine this from a more long-term perspective.

Third, in some cases, cohabitating family members were asked to monitor falls, raising the possibility that they may not have fully recognized all fall occurrences. More objective fall-recording methods should be implemented to improve accuracy. Finally, this study did not standardize PSP subtypes; future research with larger samples should compare and examine fall frequency by subtype, including typical PSP-RS, PSP-P with PD-like clinical features, and PSP-PGF with freezing of gait.

## Conclusion

This study investigated factors associated with fall frequency in patients with PSP. The results showed that the BBS was not associated with fall frequency, whereas the Mini-BESTest was associated with fall frequency. Among the Mini-BESTest subscales, anticipatory postural adjustments showed a significant association. In patients with PSP primarily characterized by postural reflex disorders, postural control ability preceding movement may be associated with fall frequency.
